# Supplementary material for: School Teachers’ Self-Reported Fear and Risk Perception during the COVID-19 Pandemic—A Nationwide Survey in Germany
Source: Int J Environ Res Public Health. 2021 Sep 1;18(17):9218. doi: 10.3390/ijerph18179218 (PMC8430488; doi:10.3390/ijerph18179218)
Supplement: Supplementary file 1 [file ijerph-18-09218-s001.zip › ijerph-1318806/ijerph-1318806-supplementary tables.pdf]

Supplementary table S1 Sources from which participants were recruited (*n=6667*).

Source	n (%)
News4Teacher	2803 (42.0)
School boards	1101 (16.5)
Education and Training Association (VBE) and the Union for Education and Training (GEW)	2173 (32.6)
German Teachers' Association	330 (5.0)
Association of Waldorf Schools	254 (3.8)
Twitter @CSozialmedizin	4 (0.1)
Montessori Association	1 (0.01)
Unknown	1 (0.01)

Supplementary table S2 Geographical distribution of the participating teachers (*n=5128*).

Zip code	n (%)
00000 – 09999	378 (7.4)
10000 – 19999	687 (13.4)
20000 – 29999	658 (12.8)
30000 – 39999	676 (13.2)
40000 – 49999	554 (10.8)
50000 – 59999	479 (9.3)
60000 – 69999	363 (7.1)
70000 – 79999	494 (9.6)
80000 – 89999	315 (6.1)
90000 – 99999	524 (10.2)

Supplementary table S3 Teachers' fear of contracting COVID-19 at school and their vaccination intention.

Variable	n (%)
**"I'm afraid contracting coronavirus at my workplace." (*n=5936)***	
I agree completely	2526 (42.6)
I agree	1807 (30.4)
Neither nor	499 (8.4)
I don't agree	434 (7.3)
I don't agree at all	670 (11.3)
**"Children can carry and transmit the virus without developing COVID-19 symptoms. Is this worrying to you?" (*n=5930*)**	
I agree completely	2749 (46.4)
I agree	1484 (25.0)
Neither nor	422 (7.1)
I don't agree	464 (7.8)
I don't agree at all	811 (13.7)
**"Do you think that schools and day-cares contribute greatly spreading of the virus?" (*n=5930*)**	
I agree completely	1952 (32.9)
I agree	1931 (32.6)
Neither nor	781 (13.2)
I don't agree	513 (8.7)
I don't agree at all	753 (12.7)

**"Do you think that children contribute greatly spreading the virus in schools and day-cares?" (*n=5930*)**	
I agree completely	1777 (30.0)
I agree	2085 (35.2)
Neither nor	843 (14.2)
I don't agree	484 (8.2)
I don't agree at all	741 (12.5)
**"Do you intent to receive a COVID-19 vaccination?" (*n=5850*)**	
Yes	4512 (77.1)
No	1019 (17.4)
Not sure yet	319 (5.5)
**"I would like that all colleagues had received a COVID-19 vaccination before returning to classroom teaching." (*n=5872)***	
I agree completely	2942 (50.1)
I agree	1004 (17.1)
Neither nor	588 (10.0)
I don't agree	332 (5.7)
I don't agree at all	1006 (17.3)

Supplementary table S4 Assessment of teacher's risk perception at work.

**Variable**	**n (%)**
**"If you agree, by whom do you feel most threatened? (Multiple answers possible)" (*n=4320*)**	
Students	4224 (97.8)
Parents	542 (12.6)
Younger colleagues (until 55 years)	1894 (43.8)
Older colleagues (> 55 years)	1308 (30.3)
I don't know	61 (1.4)
**"Are you a risk factor for the students or do the students put teachers at risk? (Multiple answers possible)" (*n=5840*)**	
Students put teachers at risk	1113 (19.1)
Teachers put students at risk	540 (9.3)
The risks come from both sides	4479 (76.7)
A high risk only exists towards older teachers (from 60/65 years)	685 (11.7)
I don't know	330 (5.7)

Supplementary table S5 Association factors for teacher's fear contracting SARS-CoV-2.

**Variable (n=6753)**	**Odds ratio (95% CI)**	**p-value**
Age	0.98 (0.97 – 0.99)	0.0008***
Intention to get vaccinated		
Yes#	1	
No	0.02 (0.02 – 0.03)	<0.0001***
Not sure	0.17 (0.12 – 0.23)	<0.0001***
Gender		
Male#	1	
Female	1.92 (1.50 – 2.45)	<0.0001***
Diverse	0.63 (0.12 – 3.25)	0.5818
Schools		
Elementary schools#	1	
Secondary schools (Hauptschulen)	1.20 (0.66 – 2.20)	0.5442
Secondary schools (Realschulen)	1.12 (0.74 – 1.68)	0.5947
High schools	0.96 (0.68 – 1.35)	0.8014
Comprehensive schools	0.99 (0.68 – 1.45)	0.9760
Schools for children with special needs	0.63 (0.42 – 0.95)	0.0264*
Waldorf schools	0.38 (0.24 – 0.62)	<0.0001***
Montessori schools	0.57 (0.11 – 2.92)	0.5006
Boarding schools	3.17 (0.01 – 1204.81)	0.7033
Privat schools	0.79 (0.39 – 1.60)	0.5136
Language schools	3.15 (0.56 – 1.78)	0.1945
Opening schools as highest priority		
I agree completely#	1	
I agree	1.63 (1.26 – 2.09)	0.0002***
Neither nor	2.04 (1.53 – 2.72)	<0.0001***
I don't agree	4.87 (3.49 – 6.80)	<0.0001***
I don't agree at all	8.94 (4.69 – 17.05)	<0.0001***
Risk perception of getting COVID-19		
High#	1	
Moderate	0.12 (0.08 – 0.17)	<0.0001***
Little	0.03 (0.02 – 0.04)	<0.0001***
No	0.01 (0.00 – 0.02)	<0.0001***
Subjects taught		
German language, no#	1	
German language, yes	0.96 (75 – 1.23)	0.7542
Mathematics, no#	1	
Mathematics, yes	0.92 (0.72 – 1.16)	0.4611
Science, no#	1	
Science, yes	0.96 (0.76 – 1.22)	0.7263
„Science at elementary schools“, no#	1	
„Science at elementary schools “, yes	0.79 (0.60 – 1.03)	0.0840
Foreign languages, no#	1	
Foreign languages	0.80 (0.64 – 0.99)	0.0424*
Sports, no#	1	
Sports, yes	0.90 (0.69 – 1.18)	0.4593
Music, no#	1	
Music, yes	1.34 (1.00 – 1.81)	0.0501
Others, no#	1	
Others, yes	1.08 (0.88 – 1.33)	0.4447

Multivariable analysis for association factors of teacher's fear getting infected with SARS-CoV-2.

#references; p values (two-sided, paired t test) for association between references and respective answers. *p value <0.05; *** p value <0.001

Supplementary table S6 Teachers' perception towards the student's parents and COVID-19 precautions.

**Variable**	**n (%)**
**"Many parents are too careless with corona." (*n=5926*)**	
I agree completely	978 (16.5)
I agree	1812 (30.6)
Neither nor	1485 (25.1)
I don't agree	1052 (17.8)
I don't agree at all	599 (10.1)
**"Many parents only think about themselves and childcare." (*n=5925*)**	
I agree completely	1003 (16.9)
I agree	1674 (28.3)
Neither nor	1500 (25.3)
I don't agree	1180 (19.9)
I don't agree at all	568 (9.6)
**"I give in to the parents' insistence on additional hygiene measures (group divisions, setting up disinfection dispensers, etc.) ...." (*n=5820*)**	
Always	553 (9.5)
Sometimes	1140 (19.6)
Rarely	950 (16.3)
Never	727 (12.5)
I don't know	2450 (42.1)
**"The requirements regarding the implementation of the COVID-19 precautions of the state put me under pressure." (*n=5931*)**	
I agree completely	1443 (24.3)
I agree	2020 (34.1)
Neither nor	923 (15.6)
I don't agree	1225 (20.7)
I don't agree at all	320 (5.4)
**"I feel I cannot fullfil the expectations of parents and the state with regard to the implementation of the COVID-19 precautions while considering children's needs." (*n=5907*)**	
I agree completely	1077 (18.2)
I agree	1826 (30.9)
Neither nor	1502 (25.4)
I don't agree	1069 (18.1)
I don't agree at all	433 (7.3)

Supplementary table S7 Frequencies teachers are getting tested.

Variable	n (%)
**"How often do you get tested for a corona infection?" (*n=5794*)**	
Once a week	1917 (33.1)
Once a month	164 (2.8)
According to the risk situation	2441 (42.1)
Never before	1272 (22.0)

Supplementary table S8 Teachers' opinion on COVID-19 becoming seasonal.

Variable	n (%)
**"I can imagine that COVID-19 could also become a seasonal disease like influenza due to mutations in the corona virus." (*n=5712*)**	
I agree completely	1483 (26.0)
I agree	2943 (51.5)
Neither nor	983 (17.2)
I don't agree	213 (3.7)
I don't agree at all	90 (1.6)

Supplementary table S9 Teachers' preferences for vaccines. (*n=4505*)

Variable	n (%)
**"If you had the choice, which vaccine would you prefer? (Multiple answers possible)"**	
BioNTech	3249 (72.1)
Moderna	1955 (43.4)
AstraZeneca**	989 (22.0)
Janssen	110 (2.4)
Sputnik V	235 (5.2)
Johnson&Johnson	693 (15.4)
I cannot judge that	355 (7.9)
I don't mind any	1025 (22.8)

**It should be noted, that much of the survey was conducted before the sinus vein thromboses became known as an adverse event of vaccination with the AstraZeneca vaccine. As of April 1st, 2021, the Standing Committee on Vaccination (STIKO) restricted the AstraZeneca vaccine to the age group ≥ 60 years.

Supplementary table S10 Teachers' reasons for or against COVID-19 vaccination.

Variable	n (%)
**"What are the reasons you intent to get vaccinated? (Multiple answers possible)" (*n=4505*)**	
For my own safety to be protected against COVID-19	4367 (96.9)
To be able to return to work quickly	1190 (26.4)
Protection for family and friends	4123 (91.5)
Protection for students	3367 (74.7)
Protection for others	3499 (77.7)
Vaccination certificate for more freedom to travel	1141 (25.3)
To spend leisure time as per my wish	1504 (33.4)
Special rights for vaccinated people	493 (11.0)
Reduction of lockdown measures	2407 (53.4)
No reason given	7 (0.2)

“What are the reasons you intent NOT to get vaccinated? (Multiple answers possible)” (*n=1008*)	
I already had COVID-19 disease and recovered	86 (8.5)
Concerns about vaccine reaction and adverse events	769 (76.3)
Concerns about as yet unknown long-term effects	881 (87.4)
I do not trust new vaccines	525 (52.1)
Vaccines not yet properly tested	781 (77.5)
I do not trust the manufacturers	494 (49.0)
I do not believe in COVID-19	71 (7.0)
I have a pre-existing condition (contraindication/increased risk from vaccination)	95 (9.4)
I am pregnant / I am breastfeeding	31 (3.1)
No reason given	42 (4.2)

Supplementary table S11 Teachers’ opinion on compulsory vaccinations.

Variable	n (%)
**“I support compulsory vaccination against COVID-19, which is being discussed by politicians.” (*n=5782*)**	
I agree completely	1137 (19.7)
I agree	1372 (23.7)
Neither nor	973 (16.8)
I don’t agree	960 (16.6)
I don’t agree at all	1340 (23.2)
**“In general, I support compulsory vaccinations as against measles also for other infectious diseases.” (*n=5785*)**	
I agree completely	1333 (23.0)
I agree	1486 (25.7)
Neither nor	947 (16.4)
I don’t agree	834 (14.4)
I don’t agree at all	1185 (20.5)

Supplementary table S12 Teachers’ attitude towards vaccinations.

Variable	n (%)
**“Do you have regular checks to ensure that you have received all recommended vaccinations?” (*n=5772*)**	
Ja	4016 (69.6)
Nein	1756 (30.4)
**“Do you consider the recommendations of the Standing Committee on Vaccination (STIKO) in Germany to be ...” (*n=5772*)**	
Appropriate	3551 (61.5)
Too little	414 (7.2)
Exaggerated	937 (16.2)
I don’t know	870 (15.1)

Supplementary table S13 Teachers' opinion on COVID-19 vaccine allocation and school re-openings.

**Variable**	**n (%)**
**"I think a prioritized allocation of the COVID-19 vaccine is appropriate." (*n=5797*)**	
I agree completely	1622 (28.0)
I agree	2489 (43.0)
Neither nor	959 (16.5)
I don't agree	374 (6.4)
I don't agree at all	353 (6.1)
**"I think that the new classification of teachers into group - 2 (high priority) is absolutely justified." (*n=5799*)**	
I agree completely	2592 (44.7)
I agree	1640 (28.3)
Neither nor	812 (14.0)
I don't agree	351 (6.1)
I don't agree at all	404 (7.0)
**„I think that re-opening up schools is an absolute priority?" (*n=5824*)**	
I agree completely	1525 (26.2)
I agree	1544 (26.5)
Neither nor	1104 (19.0)
I don't agree	1271 (21.8)
I don't agree at all	380 (6.5)

Appendix 1

School teachers' self-reported fear and risk perception during the COVID-19 pandemic – a nationwide survey in Germany

Stefanie Weinert[1], Anja Thronicke[1,2], Maximilian Hinse[1], Friedemann Schad[2,3], Harald Matthes[1,2,4]

Survey SARS-CoV-2 Impfbereitschaft – Lehrerschaft (ImpfREAD)

Sehr geehrte Lehrerinnen, sehr geehrte Lehrer,

die COVID-19 Pandemie und die Frage nach dem Bedarf und der Dringlichkeit der Impfungen der verschiedenen Altersklassen und Berufe wird gesellschaftlich kontrovers diskutiert. Wir möchten Ihre Sicht und Meinung zur Dringlichkeit einer COVID-19 Impfung der Lehrerinnen und Lehrer an den verschiedenen Schulen erfragen. Im Rahmen dieser **bundesweiten Umfrage** wollen wir die **Impfbereitschaft und Meinung der Lehrerinnen und Lehrer** zur COVID-19-Impfung untersuchen. Dabei werden auch medizinische Parameter zur Einschätzung des Erkrankungs- und Komplikationsrisikos für COVID-19 und erfragt (Alter, Geschlecht, Vorerkrankungen). Die **Teilnahme** an dieser Umfrage erfolgt **anonym** und eine **Registrierung** ist für die Teilnahme **nicht erforderlich**.

Wir würden uns freuen, wenn Sie uns bei unserem Vorhaben unterstützen und alle Fragen beantworten. Gerne können Sie auch den Link zu dieser Umfrage an Ihre Kolleginnen und Kollegen weiterleiten.

Die Umfrage unterliegt den Regelungen der Datenschutzgesetzgebung. Alle Ihre Angaben werden anonym behandelt, d.h. es ist sichergestellt, dass Ihre Angaben nicht mit Ihrer Person in Verbindung gebracht werden können. Die Umfrage wird ca. 20 Minuten in Anspruch nehmen.

Ich möchte die Teilnehmerinformation lesen und mehr zum Datenschutz wissen.

Anhang: ImpfREAD_Teilnehmerinformation.pdf (0.22 MB)

Unter Bezugnahme auf die Teilnehmerinformation und Datenschutzerklärung (Datenschutzerklärung ist Teil der Teilnehmerinformation) willige ich ein, an der wissenschaftlichen Studie teilzunehmen.

Ja / Nein

Informieren Sie sich über COVID-19 und den Impfstoffen in dem anhängenden Dokument.

Anhang: COVID-19 Zusatzinformation.pdf (0.05 MB)

An welcher Schule unterrichten Sie?

- Grundschule
- Hauptschule
- Realschule
- Gymnasium
- Gesamtschule
- Förderschule
- Waldorfschule
- Montessori Schule
- Internat
- Privatschule
- Sprachschule

Bitte geben Sie Ihr Alter in Jahren an:

Alter ___ Jahre

Bitte geben Sie Ihr Geschlecht an:

- weiblich
- männlich
- divers

Ich befürchte mich an meinem Arbeitsplatz mit Corona zu infizieren.

- Stimme ich völlig zu
- Stimme ich zu
- Stimme ich weder zu noch nicht zu
- Stimme ich nicht zu
- Stimme ich überhaupt nicht zu

Wenn Sie zustimmen, durch wen fühlen Sie sich am meisten gefährdet? (Mehrfachnennung möglich)

- Schüler
- Eltern
- Jüngere Kollegen (bis 55 Jahren)
- Ältere Kollegen (>55 Jahre)
- Weiß Nicht

Wer ist Ihrer Meinung nach Überträger des Corona-Virus? (Mehrfachnennung möglich)

- Alle Menschen
- Symptomatische Menschen
- Asymptomatische Menschen
- Kinder
- Jugendliche
- Junge Erwachsene (18-30 Jahren)
- Erwachsene im mittleren Alter (31- 65 Jahren)
- Ältere Erwachsene (älter als 65 Jahren)
- Nur die sogenannten „Superspreader"
- Weiß Nicht

Kinder können den Corona Virus in sich tragen und übertragen ohne COVID-19 Symptome zu entwickeln. Ist dies für sie beängstigend?

- Stimme ich völlig zu
- Stimme ich zu
- Stimme ich weder zu noch nicht zu
- Stimme ich nicht zu

- Stimme ich überhaupt nicht zu

Sehen Sie sich als Gefahr den Schülern gegenüber oder gefährden die Schüler die Lehrer*innen?

- Schüler gefährden Lehrer*innen
- Lehrer*innen gefährden Schüler
- Eine Gefahr geht von beiden Seiten aus
- Eine hohe Gefahr besteht nur gegenüber älteren Lehrer*innen (ab 60/65 Jahren)
- Weiß Nicht

Glauben Sie, dass Schulen und Kitas in großem Maße zur Verbreitung des Virus beitragen?

- Stimme ich völlig zu
- Stimme ich zu
- Stimme ich weder zu noch nicht zu
- Stimme ich nicht zu
- Stimme ich überhaupt nicht zu

Glauben Sie, dass Kinder in großem Maße zur Verbreitung des Virus in Schulen und Kitas beitragen?

- Stimme ich völlig zu
- Stimme ich zu
- Stimme ich weder zu noch nicht zu
- Stimme ich nicht zu
- Stimme ich überhaupt nicht zu

Glauben Sie, dass Erzieher*innen bzw. Lehrer*innen in großem Maße zur Verbreitung des Virus in Schulen und Kitas beitragen?

- Stimme ich völlig zu
- Stimme ich zu
- Stimme ich weder zu noch nicht zu
- Stimme ich nicht zu
- Stimme ich überhaupt nicht zu

Ich mache mir grundsätzlich sehr viele Sorgen, dass ich mich mit Corona infiziere. Dieser Aussage:

Stimme ich völlig zu
- Stimme ich zu
- Stimme ich weder zu noch nicht zu
- Stimme ich nicht zu
- Stimme ich überhaupt nicht zu

Meine Kollegen machen sich sehr viele Sorgen. Dieser Aussage:

- Stimme ich völlig zu
- Stimme ich zu
- Stimme ich weder zu noch nicht zu
- Stimme ich nicht zu
- Stimme ich überhaupt nicht zu

Meine Kollegen machen mich verrückt mit ihren Sorgen sich anzustecken. Dieser Aussage:

- Stimme ich völlig zu
- Stimme ich zu
- Stimme ich weder zu noch nicht zu
- Stimme ich nicht zu
- Stimme ich überhaupt nicht zu

Viele Eltern gehen zu unvorsichtig mit dem Thema Corona um. Dieser Aussage:

- Stimme ich völlig zu
- Stimme ich zu
- Stimme ich weder zu noch nicht zu
- Stimme ich nicht zu
- Stimme ich überhaupt nicht zu

Viele Eltern denken nur an sich und die Kinderbetreuung. Dieser Aussage:

- Stimme ich völlig zu
- Stimme ich zu
- Stimme ich weder zu noch nicht zu
- Stimme ich nicht zu
- Stimme ich überhaupt nicht zu

Ich beobachte, dass Ängste und Verhaltensweisen von den Eltern auf deren Kinder übertragen werden.

- Stimme ich völlig zu
- Stimme ich zu
- Stimme ich weder zu noch nicht zu
- Stimme ich nicht zu
- Stimme ich überhaupt nicht zu

Ich beobachte, dass sich Verhaltensweisen der Kinder während der Corona-Pandemie stark verändert haben.

- Stimme ich völlig zu
- Stimme ich zu
- Stimme ich weder zu noch nicht zu
- Stimme ich nicht zu
- Stimme ich überhaupt nicht zu

Die Vorgaben bezüglich der Umsetzung des Hygienekonzeptes des Bundeslandes setzen mich unter Druck.

- Stimme ich völlig zu
- Stimme ich zu
- Stimme ich weder zu noch nicht zu
- Stimme ich nicht zu
- Stimme ich überhaupt nicht zu

Die zusätzlichen Wünsche der Eltern bezüglich des Hygienekonzeptes setzen mich unter Druck.

- Stimme ich völlig zu
- Stimme ich zu
- Stimme ich weder zu noch nicht zu
- Stimme ich nicht zu
- Stimme ich überhaupt nicht zu

Ich gebe dem Drängen der Eltern bezüglich Extra-Hygienemaßnahmen (Gruppeneinteilungen, Desinfektionsspender aufstellen etc.)...

- Stimme ich völlig zu
- Stimme ich zu
- Stimme ich weder zu noch nicht zu
- Stimme ich nicht zu
- Stimme ich überhaupt nicht zu

Ich fühle mich den Erwartungen von Eltern und der Länder hinsichtlich der Umsetzung des Hygienekonzeptes bei kindgerechter Beschulung nicht gewachsen.

- Stimme ich völlig zu
- Stimme ich zu
- Stimme ich weder zu noch nicht zu

- Stimme ich nicht zu
- Stimme ich überhaupt nicht zu

Sind Sie bereits gegen COVID-19 geimpft? (Falls ja, könnte man darauf hinweisen, dass die Fragen rückblickend beantwortet werden sollen ODER grundsätzlich nur nicht-geimpfte teilnehmen lassen?)

- Ja
- Nein

Wenn ja, waren Sie zufrieden mit dem Impfverlauf?

- Ja
- Nein

Möchten Sie sich gegen COVID-19 impfen lassen?

- Ja
- Nein
- Ich bin mir noch nicht sicher

Wenn Sie die Wahl hätten, welchen Impfstoff würden Sie bevorzugen? (Mehrfachnennung möglich)

- BioNTech/Pfizer (Comirnaty)
- Moderna
- AstraZeneca
- Sputnik V
- Johnson&Johnson
- Kann ich nicht beurteilen
- Egal

Aus welchem Grund wollen Sie sich impfen lassen? (Mehrfachnennung möglich)

- Zur eigenen Sicherheit, um gegen COVID-19 geschützt sein
- Um rasch an den Arbeitsplatz zurückkehren zu können
- Schutz für Familie und Freunde
- Schutz für die Schüler
- Schutz für Andere
- Impfpass für mehr Reisefreiheit
- Freiheiten in der Gestaltung der Freizeit
- Sonderrechte für Geimpfte
- Verringerung der Lockdown Maßnahmen
- Keinen Grund

Aus welchem Grund wollen Sie sich NICHT impfen lassen? (Mehrfachnennung möglich)

- Bereits durchgemachte COVID-19 Erkrankung und genesen
- Bedenken vor Impfreaktion und Nebenwirkungen
- Bedenken vor bisher unbekannten Langzeitfolgen
- Ich traue neuen Impfstoffen nicht
- Impfstoffe noch gar nicht richtig getestet
- Ich traue den Herstellern nicht
- Ich glaube nicht an die Erkrankung COVID-19
- Ich habe eine Vorerkrankung (Kontraindikation/erhöhtes Risiko durch die Impfung)
- Ich bin schwanger / Ich stille
- Keinen Grund

Wenn Sie bezüglich der COVID-19 Impfung noch zögern, warum? (Mehrfachnennung möglich)

- Ich zögere nicht
- Ich will nicht eine/r der Ersten sein. Die Daten zu den Nebenwirkungen sind noch zu unsicher
- Mein Zögern ist eher emotional als rational begründet
- Ich habe Zweifel, dass die Impfung unsere Situation tatsächlich verbessern wird
- Ich bin grundsätzlich skeptisch gegenüber Impfungen

- Mein Risiko für einen sehr schweren Verlauf einer COVID-19 Erkrankung erscheint mir sehr gering
- Das Risiko Nutzen Verhältnis ist mir zu gering
- Weiß nicht

Würden Sie sich sofort gegen COVID-19 impfen lassen, wenn ausreichend Impfstoff vorhanden wäre?

- Ja
- Nein
- Egal

Ich fühle mich hinsichtlich einer COVID-19 Impfung ausreichend aufgeklärt.

- Stimme völlig zu
- Stimme zu
- Stimme weder zu noch nicht zu
- Stimme nicht zu
- Stimme überhaupt nicht zu

Ich wünsche mir eine COVID-19 Impfung aller Kollegen vor der Rückkehr zum Präsenzunterricht.

- Stimme völlig zu
- Stimme zu
- Stimme weder zu noch nicht zu
- Stimme nicht zu
- Stimme überhaupt nicht zu

Glauben Sie, dass durch die COVID-19 Impfung das Risiko der Übertragung einer Corona Infektion verringert wird?

- Stimme völlig zu
- Stimme zu
- Stimme weder zu noch nicht zu
- Stimme nicht zu
- Stimme überhaupt nicht zu

Verlangen Sie von der Forschung/Medizin/Politik eine Impfung, die eine Übertragung des Corona Virus verhindert?

- Ja
- Nein
- Ist nicht möglich
- Weiß nicht

Wie könnte man die Akzeptanz für eine COVID-19 Impfung erhöhen? (Mehrfachnennung möglich)

- Aufklärung durch die Politik
- Aufklärung durch die Medien
- Aufklärung durch ärztliches Personal
- Aufklärung durch Angehörige/Freunde/Kollegen
- Weiß nicht

Glauben Sie, dass die Corona-Krise durch die Herdenimmunität noch dieses Jahr beendet werden wird?

- Sehr wahrscheinlich
- Eher wahrscheinlich
- Teils/teils
- Eher unwahrscheinlich
- Sehr unwahrscheinlich

- Weiß Nicht

Ich halte eine priorisierte Vergabe des COVID-19 Impfstoffes für angemessen.

- Stimme völlig zu
- Stimme zu
- Stimme weder zu noch nicht zu
- Stimme nicht zu
- Stimme überhaupt nicht zu

Ich halte die neue Einteilung der Lehrer*innen in Gruppe – 2 (Hohe Priorität) für gerechtfertigt.

- Stimme völlig zu
- Stimme zu
- Stimme weder zu noch nicht zu
- Stimme nicht zu
- Stimme überhaupt nicht zu

Welche Kategorie hielten Sie für Lehrer*innen und Erzieher*innen für angemessen?

- Gruppe 1 – Höchste Priorität (=med. Personal mit hohem COVID-Kontakt)
- Gruppe 2 – Hohe Priorität (= med. Personal mit gelegentlichem COVID-Kontakt)
- Gruppe 3 – Erhöhte Priorität (Polizei)
- Gruppe 4 – Ohne Priorität
- Weiß nicht

Denken Sie, dass die Öffnung der Schulen absolute Priorität hat?

- Stimme völlig zu
- Stimme zu
- Stimme weder zu noch nicht zu
- Stimme nicht zu
- Stimme überhaupt nicht zu

Welche Schulklassen sollten ihrer Meinung nach zuerst in den Präsenzunterricht zurückkehren? (Mehrfachnennung möglich)

- Schulklassen 1-3
- Schulklassen 4-6
- Schulklassen 7-11
- Abschlussklassen
- Weiß Nicht

Wie bewerten Sie die Sekundärschäden der Pandemie im Bildungsbereich?

- Sehr hoch
- Hoch
- Mäßig
- Gering
- Sehr gering
- Weiß Nicht

Durch die COVID-19 Impfung können die Kontaktbeschränkungen und die Maskenpflicht zeitnah aufgehoben werden. Dieser Aussage:

- Stimme völlig zu
- Stimme zu
- Stimme weder zu noch nicht zu
- Stimme nicht zu

- Stimme überhaupt nicht zu

Wie bewerten Sie die derzeitigen nicht-pharmakologischen Maßnahmen (Abstand **+ H**ygiene **+ A**lltag mit Maske **+ Warn-A**PP **+ L**üften **Formel)?**

- Übertrieben
- Angemessen
- Zu gering
- Weiß nicht

Wünschen Sie sich über die Corona Pandemie hinaus eine gesteigerte Hygiene der Menschen (z.B. gründliches Händewaschen)?

- Ja
- Nein
- Weiß nicht

Waren oder sind Sie an COVID-19 erkrankt?

- Ja
- Nein

Ich glaube, dass ich durch eine durchgemachte COVID-19 Erkrankung eine Immunität erhalten habe

- Stimme völlig zu
- Stimme zu
- Stimme weder zu noch nicht zu
- Stimme nicht zu
- Stimme überhaupt nicht zu

Wie oft lassen Sie sich auf eine Corona Infektion testen?

- 1x wöchentlich
- 1x monatlich
- Situativ nach Gefährdungslage
- Noch nie

Warum lassen Sie sich auf eine Corona Infektion hin testen? (Mehrfachnennung möglich)

- Ich hatte Kontakt zu einer mit Corona infizierten Person
- Ich habe typische Symptome
- Aus Sorge vor eigener COVID-19 Erkrankung
- Vorsorglich um Angehörige zu schützen
- Vorsorglich um mein Umfeld zu schützen
- Weiß Nicht

Wie schätzen Sie Ihr Risiko an COVID-19 zu erkranken ein?

- Hoch
- Mäßig
- Gering
- Kein

Wie schätzen Sie Ihr Risiko bei COVID-Erkrankung einen komplizierenden Verlauf zu erleiden?

- Sehr gering
- Gering

- Mäßig
- Erhöht
- Hoch
- Sehr hoch

Steigt Ihre Sorge sich mit Corona zu infizieren durch die Verknappung des Impfstoffes?

- Ja
- Nein
- Weiß nicht

Ich habe Sorge, dass durch die Überlastung des Gesundheitssystems die Möglichkeiten einen Arzt aufzusuchen begrenzt sind?

- Stimme völlig zu
- Stimme zu
- Stimme weder zu noch nicht zu
- Stimme nicht zu
- Stimme überhaupt nicht zu

Ich habe Sorge, dass durch die Überlastung des Gesundheitssystems die Möglichkeiten ein Krankenhaus und ggf. eine Intensivstation aufzusuchen begrenzt sind?

- Stimme völlig zu
- Stimme zu
- Stimme weder zu noch nicht zu
- Stimme nicht zu
- Stimme überhaupt nicht zu

Ich befürworte eine von der Politik diskutierte Impfpflicht gegen COVID-19.

- Stimme völlig zu
- Stimme zu
- Stimme weder zu noch nicht zu
- Stimme nicht zu
- Stimme überhaupt nicht zu

Generell bin ich für eine Impflicht (wie bei Masern auch für andere Infektionserkrankungen).

- Stimme völlig zu
- Stimme zu
- Stimme weder zu noch nicht zu
- Stimme nicht zu
- Stimme überhaupt nicht zu

Sehen Sie grundsätzlich Impfungen als Schutzmaßnahmen vor Krankheiten an?

- Ja
- Nein
- Teilweise i. Sinne einer Risiko–Nutzen Abwägung
- Weiß nicht

Wie lange liegt Ihre letzte Impfung zurück?

- Ich habe mich in den letzten 4 Wochen impfen lassen
- Ich habe mich in den letzten 6 Monaten impfen lassen
- Ich habe mich im letzten 2 Jahren impfen lassen

- Ich habe mich in den letzten 5 Jahren impfen lassen
- Weiß nicht

Lassen Sie regelmäßig überprüfen, ob Sie alle empfohlenen Impfungen erhalten haben?

- Ja
- Nein

Wünschen Sie sich regelmäßige Erinnerungen an Impftermine?

- Ja
- Nein
- Weiß nicht

Halten Sie die Empfehlungen der ständigen Impfkommission (STIKO) in D für

- Angemessen
- Zu gering
- zu übertrieben
- Weiß nicht

Halten Sie ‚Privilegien' für geimpfte Personen für angemessen?

- Ja
- Ja, teilweise (z.B. Flugverkehr/Reisen)
- Nein
- Es handelt sich nicht um Privilegien, sondern um die Rückgabe der Grundrechte
- Weiß nicht

Haben Sie sich in den letzten Jahren gegen Grippe (Influenza-Virus) impfen lassen?

- Ja
- Nein

Sehen Sie sich durch den Corona-Virus SARS-CoV-2 mehr gefährdet als durch den saisonalen Influenza Virus?

- Stimme völlig zu
- Stimme zu
- Corona- und Influenza-Virus sind gleich gefährlich
- Stimme nicht zu
- Stimme überhaupt nicht zu
- Stimme weder zu noch nicht zu

Die Grippewelle durch den Influenza Virus gefährdet auch jüngere Menschen, wo hingegen eher ältere Menschen schwer an COVID-19 erkranken. Dieser Aussage:

- Stimme völlig zu
- Stimme zu
- Stimme weder zu noch nicht zu
- Stimme nicht zu
- Stimme überhaupt nicht zu

Eher ältere Menschen sind durch die COVID-19 Erkrankung gefährdet. Dieser Aussage:

- Stimme völlig zu
- Stimme zu
- Stimme weder zu noch nicht zu
- Stimme nicht zu
- Stimme überhaupt nicht zu

Jüngere Menschen erkranken kaum schwer an COVID-19. Dieser Aussage:

- Stimme völlig zu
- Stimme zu
- Stimme weder zu noch nicht zu
- Stimme nicht zu
- Stimme überhaupt nicht zu
-

Ich kann mir vorstellen, dass auch COVID-19 durch Mutationen im Corona-Virus wie Influenza eine saisonale Erkrankung werden könnte.

- Stimme völlig zu
- Stimme zu
- Stimme weder zu noch nicht zu
- Stimme nicht zu
- Stimme überhaupt nicht zu

Meine Sorgen vor einer COVID-19 Erkrankung verstärken sich durch die neuen Corona-Mutationen.

- Stimme völlig zu
- Stimme zu
- Stimme weder zu noch nicht zu
- Stimme nicht zu
- Stimme überhaupt nicht zu

Man sollte gerade angesichts der schwer kalkulierbaren Gefahren durch neue Virusmutationen bei der Öffnung der Schulen vorsichtig vorgehen.

- Stimme völlig zu
- Stimme zu
- Stimme weder zu noch nicht zu
- Stimme nicht zu
- Stimme überhaupt nicht zu

Ich sehe die Gefahr, dass sich durch die Corona-Mutationen unbemerkt eine dritte Welle aufbaut.

- Stimme völlig zu
- Stimme zu
- Stimme weder zu noch nicht zu
- Stimme nicht zu
- Stimme überhaupt nicht zu

Halten Sie die Aussage: „Das Corona-Virus ist ein Vorwand, um die Menschen zu unterdrücken.“ für:

- Sicher wahr
- Wahrscheinlich wahr
- Nicht wahr
- Weiß Nicht

Halten Sie die Aussage: „Es gibt geheime Mächte, die die Welt steuern.“ für:

- Sicher richtig
- Wahrscheinlich richtig
- Nicht richtig
- Weiß Nicht

Man hört ja manchmal Meinungen, dass es bei den Maßnahmen gegen die Corona Pandemie um etwas ganz anderes geht als das was Politik und Medien sagen. Ist da ihrer Meinung nach etwas dran oder ist das ein unbegründeter Verdacht?

- Ist etwas dran
- Unbegründeter Verdacht
- Unentschieden
- Weiß nicht

Ich war schon gegen Corona-Maßnahmen demonstrieren. Dieser Aussage:

- Stimme völlig zu
- Stimme zu
- Stimme weder zu noch nicht zu
- Stimme nicht zu
- Stimme überhaupt nicht zu

Ich kann mir vorstellen zeitnah gegen Corona-Maßnahmen zu demonstrieren, wenn nicht bald großzügigere Lockerung in Kraft treten. Dieser Aussage:

- Stimme völlig zu
- Stimme zu
- Stimme weder zu noch nicht zu
- Stimme nicht zu
- Stimme überhaupt nicht zu

Ich denke es geschehen viele sehr wichtige Dinge in der Welt, über die die Öffentlichkeit nie informiert wird.

- Sicher nicht
- Äußerst unwahrscheinlich
- Sehr unwahrscheinlich
- Unwahrscheinlich
- Eher unwahrscheinlich
- Unentschieden
- Eher wahrscheinlich
- Wahrscheinlich
- Sehr wahrscheinlich
- Äußerst wahrscheinlich
- Sicher (100%)

Ich denke Politiker geben uns normalerweise keine Auskunft über die wahren Motive ihrer Entscheidungen.

- Sicher nicht
- Äußerst unwahrscheinlich
- Sehr unwahrscheinlich
- Unwahrscheinlich
- Eher unwahrscheinlich
- Unentschieden
- Eher wahrscheinlich
- Wahrscheinlich
- Sehr wahrscheinlich
- Äußerst wahrscheinlich
- Sicher (100%)

Ich denke Regierungsbehörden überwachen alle Bürger genau.

- Sicher nicht
- Äußerst unwahrscheinlich
- Sehr unwahrscheinlich
- Unwahrscheinlich
- Eher unwahrscheinlich
- Unentschieden
- Eher wahrscheinlich
- Wahrscheinlich
- Sehr wahrscheinlich
- Äußerst wahrscheinlich
- Sicher (100%)

Ich denke Ereignisse, die auf den ersten Blick nicht miteinander in Verbindung zu stehen scheinen, sind oft das Ergebnis geheimer Aktivitäten.

- Sicher nicht
- Äußerst unwahrscheinlich
- Sehr unwahrscheinlich
- Unwahrscheinlich
- Eher unwahrscheinlich
- Unentschieden
- Eher wahrscheinlich
- Wahrscheinlich
- Sehr wahrscheinlich
- Äußerst wahrscheinlich
- Sicher (100%)

Ich denke es gibt geheime Organisationen, die großen Einfluss auf politische Entscheidungen haben.

- Sicher nicht
- Äußerst unwahrscheinlich
- Sehr unwahrscheinlich
- Unwahrscheinlich
- Eher unwahrscheinlich
- Unentschieden
- Eher wahrscheinlich
- Wahrscheinlich
- Sehr wahrscheinlich
- Äußerst wahrscheinlich
- Sicher

Haben Sie Kinder?

- Ja
- Nein

Wie viele Kinder haben Sie?

- 1 Kind
- 2 Kinder
- 3 und mehr Kinder

Wie alt sind Ihre Kinder (Mehrfachnennung möglich)?

- Kind bis 3 Jahre
- Kind bis 6 Jahre
- Kind bis 18 Jahre
- Volljährig

Haben Sie Kinderbetreuung in Anspruch genommen?

- Ja
- Nein

Teilen Sie sich die Betreuungsarbeit?

- Ja
- Wenn ja, betreuen die Großeltern mit?
- Nein

Sind Sie alleinerziehend?

- Ja
- Nein

Bitte geben Sie Vorerkrankungen an, z.B. Diabetes mellitus, COPD, Bluthochdruck (Mehrfachnennung möglich)

Freitext

Bitte geben Sie die Postleitzahl Ihres Wohnortes an:

_____

Welche Altersstufen unterrichten Sie?

- 1.-6. Klasse
- 7.-13. Klasse
- Alle Altersstufen

Welche Fächer unterrichten Sie (Mehrfachnennung möglich)?

- Deutsch
- Mathematik
- Naturwissenschaften
- Sachkunde
- Fremdsprachen
- Sport
- Musik
- Sonstige Fächer

Fertig. Vielen Dank für Ihre Mitarbeit!

Appendix – Figure A1

School teachers' self-reported fear and risk perception during the COVID-19 pandemic – a nationwide survey in Germany

Stefanie Weinert[1], Anja Thronicke[1,2], Maximilian Hinse[1], Friedemann Schad[2,3], Harald Matthes[1,2,4]

Survey SARS-CoV-2 Impfbereitschaft – Lehrerschaft (ImpfREAD)

Sehr geehrte Lehrerinnen, sehr geehrte Lehrer,

die COVID-19 Pandemie und die Frage nach dem Bedarf und der Dringlichkeit der Impfungen der verschiedenen Altersklassen und Berufe wird gesellschaftlich kontrovers diskutiert. Wir möchten Ihre Sicht und Meinung zur Dringlichkeit einer COVID-19 Impfung der Lehrerinnen und Lehrer an den verschiedenen Schulen erfragen. Im Rahmen dieser **bundesweiten Umfrage** wollen wir die **Impfbereitschaft und Meinung der Lehrerinnen und Lehrer** zur COVID-19-Impfung untersuchen. Dabei werden auch medizinische Parameter zur Einschätzung des Erkrankungs- und Komplikationsrisikos für COVID-19 und erfragt (Alter, Geschlecht, Vorerkrankungen). Die **Teilnahme** an dieser Umfrage erfolgt **anonym** und eine **Registrierung** ist für die Teilnahme **nicht erforderlich**.

Wir würden uns freuen, wenn Sie uns bei unserem Vorhaben unterstützen und alle Fragen beantworten. Gerne können Sie auch den Link zu dieser Umfrage an Ihre Kolleginnen und Kollegen weiterleiten.

Die Umfrage unterliegt den Regelungen der Datenschutzgesetzgebung. Alle Ihre Angaben werden anonym behandelt, d.h. es ist sichergestellt, dass Ihre Angaben nicht mit Ihrer Person in Verbindung gebracht werden können. Die Umfrage wird ca. 20 Minuten in Anspruch nehmen.

Ich möchte die Teilnehmerinformation lesen und mehr zum Datenschutz wissen.
Anhang: ImpfREAD_Teilnehmerinformation.pdf (0.22 MB)

Unter Bezugnahme auf die Teilnehmerinformation und Datenschutzerklärung (Datenschutzerklärung ist Teil der Teilnehmerinformation) willige ich ein, an der wissenschaftlichen Studie teilzunehmen.

Ja / Nein

Informieren Sie sich über COVID-19 und den Impfstoffen in dem anhängenden Dokument.

Anhang: COVID-19 Zusatzinformation.pdf (0.05 MB)

An welcher Schule unterrichten Sie?

- Grundschule
- Hauptschule
- Realschule
- Gymnasium
- Gesamtschule
- Förderschule
- Waldorfschule
- Montessori Schule
- Internat
- Privatschule
- Sprachschule

Bitte geben Sie Ihr Alter in Jahren an:

Alter ___ Jahre

Bitte geben Sie Ihr Geschlecht an:

- weiblich
- männlich
- divers

Ich befürchte mich an meinem Arbeitsplatz mit Corona zu infizieren.

- Stimme ich völlig zu
- Stimme ich zu
- Stimme ich weder zu noch nicht zu
- Stimme ich nicht zu
- Stimme ich überhaupt nicht zu

Wenn Sie zustimmen, durch wen fühlen Sie sich am meisten gefährdet? (Mehrfachnennung möglich)

- Schüler
- Eltern
- Jüngere Kollegen (bis 55 Jahren)
- Ältere Kollegen (>55 Jahre)
- Weiß Nicht

Wer ist Ihrer Meinung nach Überträger des Corona-Virus? (Mehrfachnennung möglich)

- Alle Menschen
- Symptomatische Menschen
- Asymptomatische Menschen
- Kinder
- Jugendliche
- Junge Erwachsene (18-30 Jahren)
- Erwachsene im mittleren Alter (31- 65 Jahren)
- Ältere Erwachsene (älter als 65 Jahren)
- Nur die sogenannten „Superspreader“
- Weiß Nicht

Kinder können den Corona Virus in sich tragen und übertragen ohne COVID-19 Symptome zu entwickeln. Ist dies für sie beängstigend?

- Stimme ich völlig zu
- Stimme ich zu
- Stimme ich weder zu noch nicht zu
- Stimme ich nicht zu

- Stimme ich überhaupt nicht zu

Sehen Sie sich als Gefahr den Schülern gegenüber oder gefährden die Schüler die Lehrer*innen?

- Schüler gefährden Lehrer*innen
- Lehrer*innen gefährden Schüler
- Eine Gefahr geht von beiden Seiten aus
- Eine hohe Gefahr besteht nur gegenüber älteren Lehrer*innen (ab 60/65 Jahren)
- Weiß Nicht

Glauben Sie, dass Schulen und Kitas in großem Maße zur Verbreitung des Virus beitragen?

- Stimme ich völlig zu
- Stimme ich zu
- Stimme ich weder zu noch nicht zu
- Stimme ich nicht zu
- Stimme ich überhaupt nicht zu

Glauben Sie, dass Kinder in großem Maße zur Verbreitung des Virus in Schulen und Kitas beitragen?

- Stimme ich völlig zu
- Stimme ich zu
- Stimme ich weder zu noch nicht zu
- Stimme ich nicht zu
- Stimme ich überhaupt nicht zu

Glauben Sie, dass Erzieher*innen bzw. Lehrer*innen in großem Maße zur Verbreitung des Virus in Schulen und Kitas beitragen?

- Stimme ich völlig zu
- Stimme ich zu
- Stimme ich weder zu noch nicht zu
- Stimme ich nicht zu
- Stimme ich überhaupt nicht zu

Ich mache mir grundsätzlich sehr viele Sorgen, dass ich mich mit Corona infiziere. Dieser Aussage:

Stimme ich völlig zu
- Stimme ich zu
- Stimme ich weder zu noch nicht zu
- Stimme ich nicht zu
- Stimme ich überhaupt nicht zu

Meine Kollegen machen sich sehr viele Sorgen. Dieser Aussage:

- Stimme ich völlig zu
- Stimme ich zu
- Stimme ich weder zu noch nicht zu
- Stimme ich nicht zu
- Stimme ich überhaupt nicht zu

Meine Kollegen machen mich verrückt mit ihren Sorgen sich anzustecken. Dieser Aussage:

- Stimme ich völlig zu
- Stimme ich zu
- Stimme ich weder zu noch nicht zu
- Stimme ich nicht zu
- Stimme ich überhaupt nicht zu

Viele Eltern gehen zu unvorsichtig mit dem Thema Corona um. Dieser Aussage:

- Stimme ich völlig zu
- Stimme ich zu
- Stimme ich weder zu noch nicht zu
- Stimme ich nicht zu
- Stimme ich überhaupt nicht zu

Viele Eltern denken nur an sich und die Kinderbetreuung. Dieser Aussage:

- Stimme ich völlig zu
- Stimme ich zu
- Stimme ich weder zu noch nicht zu
- Stimme ich nicht zu
- Stimme ich überhaupt nicht zu

Ich beobachte, dass Ängste und Verhaltensweisen von den Eltern auf deren Kinder übertragen werden.

- Stimme ich völlig zu
- Stimme ich zu
- Stimme ich weder zu noch nicht zu
- Stimme ich nicht zu
- Stimme ich überhaupt nicht zu

Ich beobachte, dass sich Verhaltensweisen der Kinder während der Corona-Pandemie stark verändert haben.

- Stimme ich völlig zu
- Stimme ich zu
- Stimme ich weder zu noch nicht zu
- Stimme ich nicht zu
- Stimme ich überhaupt nicht zu

Die Vorgaben bezüglich der Umsetzung des Hygienekonzeptes des Bundeslandes setzen mich unter Druck.

- Stimme ich völlig zu
- Stimme ich zu
- Stimme ich weder zu noch nicht zu
- Stimme ich nicht zu
- Stimme ich überhaupt nicht zu

Die zusätzlichen Wünsche der Eltern bezüglich des Hygienekonzeptes setzen mich unter Druck.

- Stimme ich völlig zu
- Stimme ich zu
- Stimme ich weder zu noch nicht zu
- Stimme ich nicht zu
- Stimme ich überhaupt nicht zu

Ich gebe dem Drängen der Eltern bezüglich Extra-Hygienemaßnahmen (Gruppeneinteilungen, Desinfektionsspender aufstellen etc.)...

- Stimme ich völlig zu
- Stimme ich zu
- Stimme ich weder zu noch nicht zu
- Stimme ich nicht zu
- Stimme ich überhaupt nicht zu

Ich fühle mich den Erwartungen von Eltern und der Länder hinsichtlich der Umsetzung des Hygienekonzeptes bei kindgerechter Beschulung nicht gewachsen.

- Stimme ich völlig zu
- Stimme ich zu
- Stimme ich weder zu noch nicht zu

- Stimme ich nicht zu
- Stimme ich überhaupt nicht zu

Sind Sie bereits gegen COVID-19 geimpft? (Falls ja, könnte man darauf hinweisen, dass die Fragen rückblickend beantwortet werden sollen ODER grundsätzlich nur nicht-geimpfte teilnehmen lassen?)

- Ja
- Nein

Wenn ja, waren Sie zufrieden mit dem Impfverlauf?

- Ja
- Nein

Möchten Sie sich gegen COVID-19 impfen lassen?

- Ja
- Nein
- Ich bin mir noch nicht sicher

Wenn Sie die Wahl hätten, welchen Impfstoff würden Sie bevorzugen? (Mehrfachnennung möglich)

- BioNTech/Pfizer (Comirnaty)
- Moderna
- AstraZeneca
- Sputnik V
- Johnson&Johnson
- Kann ich nicht beurteilen
- Egal

Aus welchem Grund wollen Sie sich impfen lassen? (Mehrfachnennung möglich)

- Zur eigenen Sicherheit, um gegen COVID-19 geschützt sein
- Um rasch an den Arbeitsplatz zurückkehren zu können
- Schutz für Familie und Freunde
- Schutz für die Schüler
- Schutz für Andere
- Impfpass für mehr Reisefreiheit
- Freiheiten in der Gestaltung der Freizeit
- Sonderrechte für Geimpfte
- Verringerung der Lockdown Maßnahmen
- Keinen Grund

Aus welchem Grund wollen Sie sich NICHT impfen lassen? (Mehrfachnennung möglich)

- Bereits durchgemachte COVID-19 Erkrankung und genesen
- Bedenken vor Impfreaktion und Nebenwirkungen
- Bedenken vor bisher unbekannten Langzeitfolgen
- Ich traue neuen Impfstoffen nicht
- Impfstoffe noch gar nicht richtig getestet
- Ich traue den Herstellern nicht
- Ich glaube nicht an die Erkrankung COVID-19
- Ich habe eine Vorerkrankung (Kontraindikation/erhöhtes Risiko durch die Impfung)
- Ich bin schwanger / Ich stille
- Keinen Grund

Wenn Sie bezüglich der COVID-19 Impfung noch zögern, warum? (Mehrfachnennung möglich)

- Ich zögere nicht
- Ich will nicht eine/r der Ersten sein. Die Daten zu den Nebenwirkungen sind noch zu unsicher
- Mein Zögern ist eher emotional als rational begründet
- Ich habe Zweifel, dass die Impfung unsere Situation tatsächlich verbessern wird
- Ich bin grundsätzlich skeptisch gegenüber Impfungen

- Mein Risiko für einen sehr schweren Verlauf einer COVID-19 Erkrankung erscheint mir sehr gering
- Das Risiko Nutzen Verhältnis ist mir zu gering
- Weiß nicht

Würden Sie sich sofort gegen COVID-19 impfen lassen, wenn ausreichend Impfstoff vorhanden wäre?

- Ja
- Nein
- Egal

Ich fühle mich hinsichtlich einer COVID-19 Impfung ausreichend aufgeklärt.

- Stimme völlig zu
- Stimme zu
- Stimme weder zu noch nicht zu
- Stimme nicht zu
- Stimme überhaupt nicht zu

Ich wünsche mir eine COVID-19 Impfung aller Kollegen vor der Rückkehr zum Präsenzunterricht.

- Stimme völlig zu
- Stimme zu
- Stimme weder zu noch nicht zu
- Stimme nicht zu
- Stimme überhaupt nicht zu

Glauben Sie, dass durch die COVID-19 Impfung das Risiko der Übertragung einer Corona Infektion verringert wird?

- Stimme völlig zu
- Stimme zu
- Stimme weder zu noch nicht zu
- Stimme nicht zu
- Stimme überhaupt nicht zu

Verlangen Sie von der Forschung/Medizin/Politik eine Impfung, die eine Übertragung des Corona Virus verhindert?

- Ja
- Nein
- Ist nicht möglich
- Weiß nicht

Wie könnte man die Akzeptanz für eine COVID-19 Impfung erhöhen? (Mehrfachnennung möglich)

- Aufklärung durch die Politik
- Aufklärung durch die Medien
- Aufklärung durch ärztliches Personal
- Aufklärung durch Angehörige/Freunde/Kollegen
- Weiß nicht

Glauben Sie, dass die Corona-Krise durch die Herdenimmunität noch dieses Jahr beendet werden wird?

- Sehr wahrscheinlich
- Eher wahrscheinlich
- Teils/teils
- Eher unwahrscheinlich
- Sehr unwahrscheinlich

- Weiß Nicht

Ich halte eine priorisierte Vergabe des COVID-19 Impfstoffes für angemessen.

- Stimme völlig zu
- Stimme zu
- Stimme weder zu noch nicht zu
- Stimme nicht zu
- Stimme überhaupt nicht zu

Ich halte die neue Einteilung der Lehrer*innen in Gruppe – 2 (Hohe Priorität) für gerechtfertigt.

- Stimme völlig zu
- Stimme zu
- Stimme weder zu noch nicht zu
- Stimme nicht zu
- Stimme überhaupt nicht zu

Welche Kategorie hielten Sie für Lehrer*innen und Erzieher*innen für angemessen?

- Gruppe 1 – Höchste Priorität (=med. Personal mit hohem COVID-Kontakt)
- Gruppe 2 – Hohe Priorität (= med. Personal mit gelegentlichem COVID-Kontakt)
- Gruppe 3 – Erhöhte Priorität (Polizei)
- Gruppe 4 – Ohne Priorität
- Weiß nicht

Denken Sie, dass die Öffnung der Schulen absolute Priorität hat?

- Stimme völlig zu
- Stimme zu
- Stimme weder zu noch nicht zu
- Stimme nicht zu
- Stimme überhaupt nicht zu

Welche Schulklassen sollten ihrer Meinung nach zuerst in den Präsenzunterricht zurückkehren? (Mehrfachnennung möglich)

- Schulklassen 1-3
- Schulklassen 4-6
- Schulklassen 7-11
- Abschlussklassen
- Weiß Nicht

Wie bewerten Sie die Sekundärschäden der Pandemie im Bildungsbereich?

- Sehr hoch
- Hoch
- Mäßig
- Gering
- Sehr gering
- Weiß Nicht

Durch die COVID-19 Impfung können die Kontaktbeschränkungen und die Maskenpflicht zeitnah aufgehoben werden. Dieser Aussage:

- Stimme völlig zu
- Stimme zu
- Stimme weder zu noch nicht zu
- Stimme nicht zu

- Stimme überhaupt nicht zu

Wie bewerten Sie die derzeitigen nicht-pharmakologischen Maßnahmen (Abstand **+ H**ygiene **+ A**lltag mit Maske **+ Warn-A**PP **+ L**üften **Formel)?**

- Übertrieben
- Angemessen
- Zu gering
- Weiß nicht

Wünschen Sie sich über die Corona Pandemie hinaus eine gesteigerte Hygiene der Menschen (z.B. gründliches Händewaschen)?

- Ja
- Nein
- Weiß nicht

Waren oder sind Sie an COVID-19 erkrankt?

- Ja
- Nein

Ich glaube, dass ich durch eine durchgemachte COVID-19 Erkrankung eine Immunität erhalten habe

- Stimme völlig zu
- Stimme zu
- Stimme weder zu noch nicht zu
- Stimme nicht zu
- Stimme überhaupt nicht zu

Wie oft lassen Sie sich auf eine Corona Infektion testen?

- 1x wöchentlich
- 1x monatlich
- Situativ nach Gefährdungslage
- Noch nie

Warum lassen Sie sich auf eine Corona Infektion hin testen? (Mehrfachnennung möglich)

- Ich hatte Kontakt zu einer mit Corona infizierten Person
- Ich habe typische Symptome
- Aus Sorge vor eigener COVID-19 Erkrankung
- Vorsorglich um Angehörige zu schützen
- Vorsorglich um mein Umfeld zu schützen
- Weiß Nicht

Wie schätzen Sie Ihr Risiko an COVID-19 zu erkranken ein?

- Hoch
- Mäßig
- Gering
- Kein

Wie schätzen Sie Ihr Risiko bei COVID-Erkrankung einen komplizierenden Verlauf zu erleiden?

- Sehr gering
- Gering

- Mäßig
- Erhöht
- Hoch
- Sehr hoch

Steigt Ihre Sorge sich mit Corona zu infizieren durch die Verknappung des Impfstoffes?

- Ja
- Nein
- Weiß nicht

Ich habe Sorge, dass durch die Überlastung des Gesundheitssystems die Möglichkeiten einen Arzt aufzusuchen begrenzt sind?

- Stimme völlig zu
- Stimme zu
- Stimme weder zu noch nicht zu
- Stimme nicht zu
- Stimme überhaupt nicht zu

Ich habe Sorge, dass durch die Überlastung des Gesundheitssystems die Möglichkeiten ein Krankenhaus und ggf. eine Intensivstation aufzusuchen begrenzt sind?

- Stimme völlig zu
- Stimme zu
- Stimme weder zu noch nicht zu
- Stimme nicht zu
- Stimme überhaupt nicht zu

Ich befürworte eine von der Politik diskutierte Impfpflicht gegen COVID-19.

- Stimme völlig zu
- Stimme zu
- Stimme weder zu noch nicht zu
- Stimme nicht zu
- Stimme überhaupt nicht zu

Generell bin ich für eine Impflicht (wie bei Masern auch für andere Infektionserkrankungen).

- Stimme völlig zu
- Stimme zu
- Stimme weder zu noch nicht zu
- Stimme nicht zu
- Stimme überhaupt nicht zu

Sehen Sie grundsätzlich Impfungen als Schutzmaßnahmen vor Krankheiten an?

- Ja
- Nein
- Teilweise i. Sinne einer Risiko–Nutzen Abwägung
- Weiß nicht

Wie lange liegt Ihre letzte Impfung zurück?

- Ich habe mich in den letzten 4 Wochen impfen lassen
- Ich habe mich in den letzten 6 Monaten impfen lassen
- Ich habe mich im letzten 2 Jahren impfen lassen

- Ich habe mich in den letzten 5 Jahren impfen lassen
- Weiß nicht

Lassen Sie regelmäßig überprüfen, ob Sie alle empfohlenen Impfungen erhalten haben?

- Ja
- Nein

Wünschen Sie sich regelmäßige Erinnerungen an Impftermine?

- Ja
- Nein
- Weiß nicht

Halten Sie die Empfehlungen der ständigen Impfkommission (STIKO) in D für

- Angemessen
- Zu gering
- zu übertrieben
- Weiß nicht

Halten Sie ‚Privilegien' für geimpfte Personen für angemessen?

- Ja
- Ja, teilweise (z.B. Flugverkehr/Reisen)
- Nein
- Es handelt sich nicht um Privilegien, sondern um die Rückgabe der Grundrechte
- Weiß nicht

Haben Sie sich in den letzten Jahren gegen Grippe (Influenza-Virus) impfen lassen?

- Ja
- Nein

Sehen Sie sich durch den Corona-Virus SARS-CoV-2 mehr gefährdet als durch den saisonalen Influenza Virus?

- Stimme völlig zu
- Stimme zu
- Corona- und Influenza-Virus sind gleich gefährlich
- Stimme nicht zu
- Stimme überhaupt nicht zu
- Stimme weder zu noch nicht zu

Die Grippewelle durch den Influenza Virus gefährdet auch jüngere Menschen, wo hingegen eher ältere Menschen schwer an COVID-19 erkranken. Dieser Aussage:

- Stimme völlig zu
- Stimme zu
- Stimme weder zu noch nicht zu
- Stimme nicht zu
- Stimme überhaupt nicht zu

Eher ältere Menschen sind durch die COVID-19 Erkrankung gefährdet. Dieser Aussage:

- Stimme völlig zu
- Stimme zu
- Stimme weder zu noch nicht zu
- Stimme nicht zu
- Stimme überhaupt nicht zu

Jüngere Menschen erkranken kaum schwer an COVID-19. Dieser Aussage:

- Stimme völlig zu
- Stimme zu
- Stimme weder zu noch nicht zu
- Stimme nicht zu
- Stimme überhaupt nicht zu
-

Ich kann mir vorstellen, dass auch COVID-19 durch Mutationen im Corona-Virus wie Influenza eine saisonale Erkrankung werden könnte.

- Stimme völlig zu
- Stimme zu
- Stimme weder zu noch nicht zu
- Stimme nicht zu
- Stimme überhaupt nicht zu

Meine Sorgen vor einer COVID-19 Erkrankung verstärken sich durch die neuen Corona-Mutationen.

- Stimme völlig zu
- Stimme zu
- Stimme weder zu noch nicht zu
- Stimme nicht zu
- Stimme überhaupt nicht zu

Man sollte gerade angesichts der schwer kalkulierbaren Gefahren durch neue Virusmutationen bei der Öffnung der Schulen vorsichtig vorgehen.

- Stimme völlig zu
- Stimme zu
- Stimme weder zu noch nicht zu
- Stimme nicht zu
- Stimme überhaupt nicht zu

Ich sehe die Gefahr, dass sich durch die Corona-Mutationen unbemerkt eine dritte Welle aufbaut.

- Stimme völlig zu
- Stimme zu
- Stimme weder zu noch nicht zu
- Stimme nicht zu
- Stimme überhaupt nicht zu

Halten Sie die Aussage: „Das Corona-Virus ist ein Vorwand, um die Menschen zu unterdrücken." für:

- Sicher wahr
- Wahrscheinlich wahr
- Nicht wahr
- Weiß Nicht

Halten Sie die Aussage: „Es gibt geheime Mächte, die die Welt steuern." für:

- Sicher richtig
- Wahrscheinlich richtig
- Nicht richtig
- Weiß Nicht

Man hört ja manchmal Meinungen, dass es bei den Maßnahmen gegen die Corona Pandemie um etwas ganz anderes geht als das was Politik und Medien sagen. Ist da ihrer Meinung nach etwas dran oder ist das ein unbegründeter Verdacht?

- Ist etwas dran
- Unbegründeter Verdacht
- Unentschieden
- Weiß nicht

Ich war schon gegen Corona-Maßnahmen demonstrieren. Dieser Aussage:

- Stimme völlig zu
- Stimme zu
- Stimme weder zu noch nicht zu
- Stimme nicht zu
- Stimme überhaupt nicht zu

Ich kann mir vorstellen zeitnah gegen Corona-Maßnahmen zu demonstrieren, wenn nicht bald großzügigere Lockerung in Kraft treten. Dieser Aussage:

- Stimme völlig zu
- Stimme zu
- Stimme weder zu noch nicht zu
- Stimme nicht zu
- Stimme überhaupt nicht zu

Ich denke es geschehen viele sehr wichtige Dinge in der Welt, über die die Öffentlichkeit nie informiert wird.

- Sicher nicht
- Äußerst unwahrscheinlich
- Sehr unwahrscheinlich
- Unwahrscheinlich
- Eher unwahrscheinlich
- Unentschieden
- Eher wahrscheinlich
- Wahrscheinlich
- Sehr wahrscheinlich
- Äußerst wahrscheinlich
- Sicher (100%)

Ich denke Politiker geben uns normalerweise keine Auskunft über die wahren Motive ihrer Entscheidungen.

- Sicher nicht
- Äußerst unwahrscheinlich
- Sehr unwahrscheinlich
- Unwahrscheinlich
- Eher unwahrscheinlich
- Unentschieden
- Eher wahrscheinlich
- Wahrscheinlich
- Sehr wahrscheinlich
- Äußerst wahrscheinlich
- Sicher (100%)

Ich denke Regierungsbehörden überwachen alle Bürger genau.

- Sicher nicht
- Äußerst unwahrscheinlich
- Sehr unwahrscheinlich
- Unwahrscheinlich
- Eher unwahrscheinlich
- Unentschieden
- Eher wahrscheinlich
- Wahrscheinlich
- Sehr wahrscheinlich
- Äußerst wahrscheinlich
- Sicher (100%)

Ich denke Ereignisse, die auf den ersten Blick nicht miteinander in Verbindung zu stehen scheinen, sind oft das Ergebnis geheimer Aktivitäten.

- Sicher nicht
- Äußerst unwahrscheinlich
- Sehr unwahrscheinlich
- Unwahrscheinlich
- Eher unwahrscheinlich
- Unentschieden
- Eher wahrscheinlich
- Wahrscheinlich
- Sehr wahrscheinlich
- Äußerst wahrscheinlich
- Sicher (100%)

Ich denke es gibt geheime Organisationen, die großen Einfluss auf politische Entscheidungen haben.

- Sicher nicht
- Äußerst unwahrscheinlich
- Sehr unwahrscheinlich
- Unwahrscheinlich
- Eher unwahrscheinlich
- Unentschieden
- Eher wahrscheinlich
- Wahrscheinlich
- Sehr wahrscheinlich
- Äußerst wahrscheinlich
- Sicher

Haben Sie Kinder?

- Ja
- Nein

Wie viele Kinder haben Sie?

- 1 Kind
- 2 Kinder
- 3 und mehr Kinder

Wie alt sind Ihre Kinder (Mehrfachnennung möglich)?

- Kind bis 3 Jahre
- Kind bis 6 Jahre
- Kind bis 18 Jahre
- Volljährig

Haben Sie Kinderbetreuung in Anspruch genommen?

- Ja
- Nein

Teilen Sie sich die Betreuungsarbeit?

- Ja
- Wenn ja, betreuen die Großeltern mit?
- Nein

Sind Sie alleinerziehend?

- Ja
- Nein

Bitte geben Sie Vorerkrankungen an, z.B. Diabetes mellitus, COPD, Bluthochdruck (Mehrfachnennung möglich)

Freitext

Bitte geben Sie die Postleitzahl Ihres Wohnortes an:

_____

Welche Altersstufen unterrichten Sie?

- 1.-6. Klasse
- 7.-13. Klasse
- Alle Altersstufen

Welche Fächer unterrichten Sie (Mehrfachnennung möglich)?

- Deutsch
- Mathematik
- Naturwissenschaften
- Sachkunde
- Fremdsprachen
- Sport
- Musik
- Sonstige Fächer

Fertig. Vielen Dank für Ihre Mitarbeit!
